# FixJ family regulator AcfR of *Azorhizobium caulinodans* is involved in symbiosis with the host plant

**DOI:** 10.1186/s12866-021-02138-w

**Published:** 2021-03-09

**Authors:** Wei Liu, Xue Bai, Yan Li, Haikun Zhang, Xiaoke Hu

**Affiliations:** 1 Key Laboratory of Coastal Biology and Bioresource Utilization, Yantai Institute of Coastal Zone Research, Chinese Academy of Sciences, Yantai, 264003 China; 2 Laboratory for Marine Biology and Biotechnology, Qingdao National Laboratory for Marine Science and Technology, Qingdao, 266237 China; 3 Center for Ocean Mega-Science, Chinese Academy of Sciences, Qingdao, China

**Keywords:** Two-component response regulator, REC domain, HTH_LuxR domain, Bacterial motility, Exopolysaccharides, Competitive nodulation

## Abstract

**Background:**

A wide variety of bacterial adaptative responses to environmental conditions are mediated by signal transduction pathways. Two-component signal transduction systems are one of the predominant means used by bacteria to sense the signals of the host plant and adjust their interaction behaviour. A total of seven open reading frames have been identified as putative two-component response regulators in the gram-negative nitrogen-fixing bacteria *Azorhizobium caulinodans* ORS571. However, the biological functions of these response regulators in the symbiotic interactions between *A. caulinodans* ORS571 and the host plant *Sesbania rostrata* have not been elucidated to date.

**Results:**

In this study, we identified and investigated a two-component response regulator, AcfR, with a phosphorylatable N-terminal REC (receiver) domain and a C-terminal HTH (helix-turn-helix) LuxR DNA-binding domain in *A. caulinodans* ORS571. Phylogenetic analysis showed that AcfR possessed close evolutionary relationships with NarL/FixJ family regulators. In addition, six histidine kinases containing HATPase_c and HisKA domains were predicted to interact with AcfR. Furthermore, the biological function of AcfR in free-living and symbiotic conditions was elucidated by comparing the wild-type strain and the Δ*acfR* mutant strain. In the free-living state, the cell motility behaviour and exopolysaccharide production of the Δ*acfR* mutant were significantly reduced compared to those of the wild-type strain. In the symbiotic state, the Δ*acfR* mutant showed a competitive nodule defect on the stems and roots of the host plant, suggesting that AcfR can provide *A. caulinodans* with an effective competitive ability for symbiotic nodulation.

**Conclusions:**

Our results showed that AcfR, as a response regulator, regulates numerous phenotypes of *A. caulinodans* under the free-living conditions and in symbiosis with the host plant. The results of this study help to elucidate the involvement of a REC + HTH_LuxR two-component response regulator in the *Rhizobium*-host plant interaction.

**Supplementary Information:**

The online version contains supplementary material available at 10.1186/s12866-021-02138-w.

## Background

The alphaproteobacterium *Azorhizobium caulinodans* ORS571, as a gram-negative nitrogen-fixing bacterium, has the dual ability to fix nitrogen both under free-living conditions and in a symbiotic interaction with the tropical legume *Sesbania rostrata*, which forms both stem nodules and root nodules [1]. The nitrogen-fixing symbiosis of soil bacteria with legume plants is a multistep process that involves an exchange of signals between compatible partners. Host plants signal compounds, which are released by induced rhizobial cells, induce the curling of root hairs. Next, rhizobial cells attach to root hairs and begin to infect root tissue. Subsequently, bacterial cells are released into plant cells, where they divide into bacteroids that will eventually return atmospheric nitrogen to ammonia [2]. The interaction between rhizobia and the host is initiated by a complex molecular dialogue.

Bacteria possess various signalling systems that enable them to sense and respond to diverse signals from external environmental conditions. Two-component system (TCS), a major type of cellular signal transduction system, are widely used by bacteria to sense and adjust their behaviour according to environmental changes [3]. TCS plays major role in a wide range of adaptive mechanisms, such as host-pathogen interactions, symbiotic interactions, intracellular signaling, metabolism, notably in response to stress conditions, and motility [4]. The canonical TCS comprises a sensor histidine kinase (HK) and a cytoplasmic response regulator (RR). Typical HKs contain a transmembrane domain and intracellular domain. Generally, the intracellular component has two obvious domains: the histidine-associated ATPase, C-terminal (HATPase_c) and the histidine kinase acceptor (HisKA). HK responds to received signals or stimuli and undergoes autophosphorylation on a conserved histidine residue. Next, the phosphate group is transferred to a conserved aspartate in the N-terminal receiver domain of the RR [5]. Phosphorylation of the RR occurs within the receiver domain and typically leads to cellular change by activating an output domain. RRs are classified into different groups according to protein domain organization. The LuxR family RRs contain a C-terminal HTH (helix-turn-helix) domain of approximately 65 amino acids, which can be activated by different mechanisms. QS (quorum sensing) LuxR regulators (such as *Vibrio fischeri* luxR and *Agrobacterium tumefaciens* traR) are activated when they bind to N-acyl homoserine lactones [6, 7]. The NarL/FixJ family RRs are the second most abundant family of bacteria [8] and have a typical HTH DNA-binding output domain that is similar to the QS LuxR family. *Sinorhizobium meliloti* FixJ [9], *Escherichia coli* NarL [10], UhpA [11], and *Enterobacteria* RcsB belong to this category. Other types of RRs contain autonomous effector domains but do not have a regulatory domain (GerE) [12] or multiple ligand-binding domains (MalT) [13].

In the symbiotic bacterium *S. meliloti*, FixJ contains a phosphorylatable N-terminal REC domain and a C-terminal HTH_LuxR DNA-binding domain. FixJ, the oxygen-sensitive two-component response regulator, plays a pivotal role in symbiotic nitrogen fixation in *S. meliloti*. FixJ is a positive activator required for the regulation of nitrogen-fixing gene transcription [9]. Genome analysis of *A. caulinodans* ORS571 (https://www.ncbi.nlm.nih.gov/) suggested the existence of a FixJ family response regulator AcfR (*A.* *caulinodans* fixJ family regulator) that contains a REC domain in the N-terminus and an HTH_LuxR domain in the C-terminus. In this work, we elucidated the potential role played by AcfR in *A. caulinodans* under free-living and symbiotic conditions.

## Results

### AcfR is a REC + HTH_LuxR response regulator of the NarL/FixJ family

**Fig. 1:**
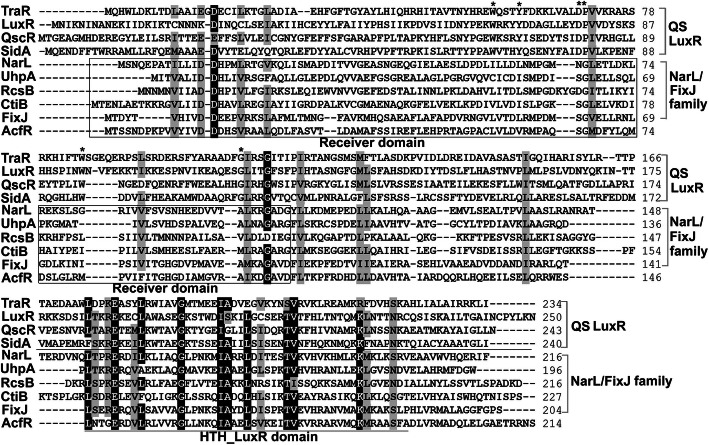
Multiple sequences aligment of AcfR and other HTH_LuxR response regulators of LuxR/FixJ subfamily. The selected QS LuxR-type sequences include *A. tumefaciens* TraR, *V. fischeri* LuxR, *Pseudomonas aeruginosa* QscR, and *Salmonella enterica* SidA. The selected NarL/FixJ family regulators include *E. coli* NarL and RcsB, *Salmonella typhimurium* UhpA, *Methylacidiphilum infernorum* CitB, and *S. meliloti* FixJ. Alignment was performed using BioEdit software. Completely conserved amino acids are shown on a black background. 80% conservation are indicated by a dark-grey background. Asterisks indicate six conserved residues in autoinducer binding domains of AHL-QS LuxRs, and the C terminal HTH domain is underlined. The receiver domain is shown in a rectangular box

To identify AcfR (WP_012168725), we analysed the domain architecture and phylogenetic relationship of AcfR and some proteins containing an HTH DNA-binding domain. First, the protein sequence was entered in SMART (http://smart.embl.de/), which indicated that it contains an N-terminal signal receiver domain REC (SM00448) and an HTH_LuxR DNA-binding domain (SM00421) (Supplemental Fig. 1a). Second, the amino-acid sequences of AcfR and some well-studied proteins containing the HTH_LuxR DNA-binding domain were aligned by BioEdit software. As shown in Fig. 1, LuxR-type proteins contained highly conserved HTH_LuxR domains and C-terminal autoinducer-binding domains (six conserved amino acid residues). However, AcfR and the other types of HTH_LuxR proteins (FixJ, NarL, UhpA, RcsB, and CitB) contain the REC domain and HTH_LuxR domain.

**Fig. 2:**
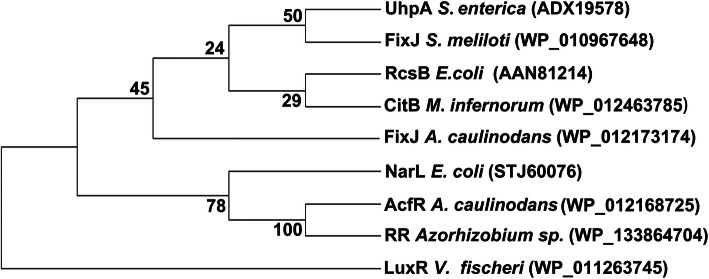
Phylogenetic analysis of AcfR and proteins with conserved HTH_LuxR and REC domains. The phylogenetic trees were constructed based on the amino acid sequence neighbor-joining algorithm by the MEG-X program, and the bootstrap test carried out with 1000 replicates. LuxR of *Vibrio fischeri* was selected as the outgroup the tree. Bootstrap values are included at the tree nodes

**Fig. 3:**
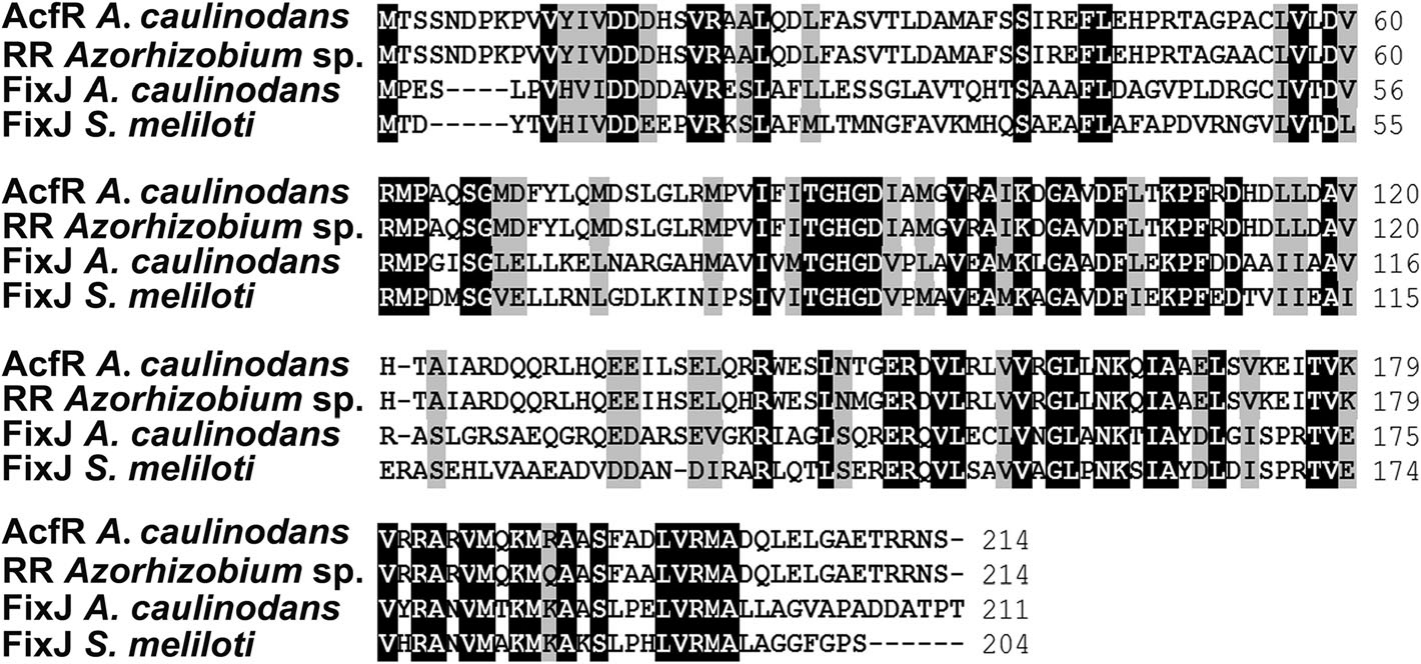
Comparison of amino acid sequences. Alignment of AcfR and FixJ family response regulator (RR) of *Azorhizobium* sp., FixJ of *S. meliloti* and *A. caulinodans* was performed using BioEdit software. Completely conserved amino acids are shown on a black background. 80% residue conservation are indicated by a grey background

Furthermore, the evolutionary relationship between AcfR and several proteins that contain REC + HTH_LuxR domains was analysed. For phylogenetic reconstruction, these proteins were aligned with the ClustalW program and the phylogenetic tree was created with MEGA-X software by the neighbor-joining method. As shown in Fig. 2, AcfR was grouped into a cluster with NarL/FixJ family response regulators. The typical QS regulator LuxR of *Vibrio fischeri* served as an outgroup. These NarL/FixJ family proteins shared the signal receiver domain REC in the N-terminus and the HTH_LuxR domain in the C-terminus. Furthermore, the amino acid sequences of AcfR and FixJ family regulators were aligned (Fig. 3). Amino acid sequence analysis showed that the similarity between AcfR and response regulator (RR) of *Azorhizobium* sp. (WP_133864704), FixJ of *S. meliloti* and *A. caulinodans* ORS571 was 97.20, 41.20, 44.02%, respectively. These results suggested that AcfR belongs to the FixJ family of two-component response regulators.

To elucidate understand the biological function of AcfR, a protein-protein interaction network was constructed using the STRING database (https://string-db.org/) [14]. As shown in Supplementary Fig. 1b, a total of 8 proteins were predicted to interact with AcfR in the genome of *A. caulinodans*, including FixL, two AAA family ATPase proteins, two PAS domain S-box proteins, a two-component sensor histidine kinase, a GHKL domain-containing protein, and a magnesium-translocating P-type ATPase. Interestingly, six of eight proteins were predicted to be histidine kinases with HisKA and HATPase_c domains (Supplementary Fig. 2). These results suggested that AcfR, as an RR of the two-component regulatory system, may be involved in histidine kinase signal transduction.

### *acfR* mutation does not affect bacterial growth

To further characterize the regulatory function of AcfR, we generated an *acfR* deletion mutant strain (Δ*acfR*) of *A. caulinodans* by gene homologous recombination and constructed its complemented strain (Δ*acfR-C*). To confirm whether this mutation affects bacterial growth under normal conditions, the dynamic growth curves of the wild-type (WT), mutant, and complemented strains were tested at various stages of growth. The results indicated that the relative growth rates of the mutant and complemented strains were not significantly different from that of the WT strain (Supplementary Fig. 3), indicating that deletion of the *acfR* gene did not significantly affect the normal growth of bacteria.

### AcfR regulated motility and exopolysaccharide production in free-living state

**Fig. 4:**
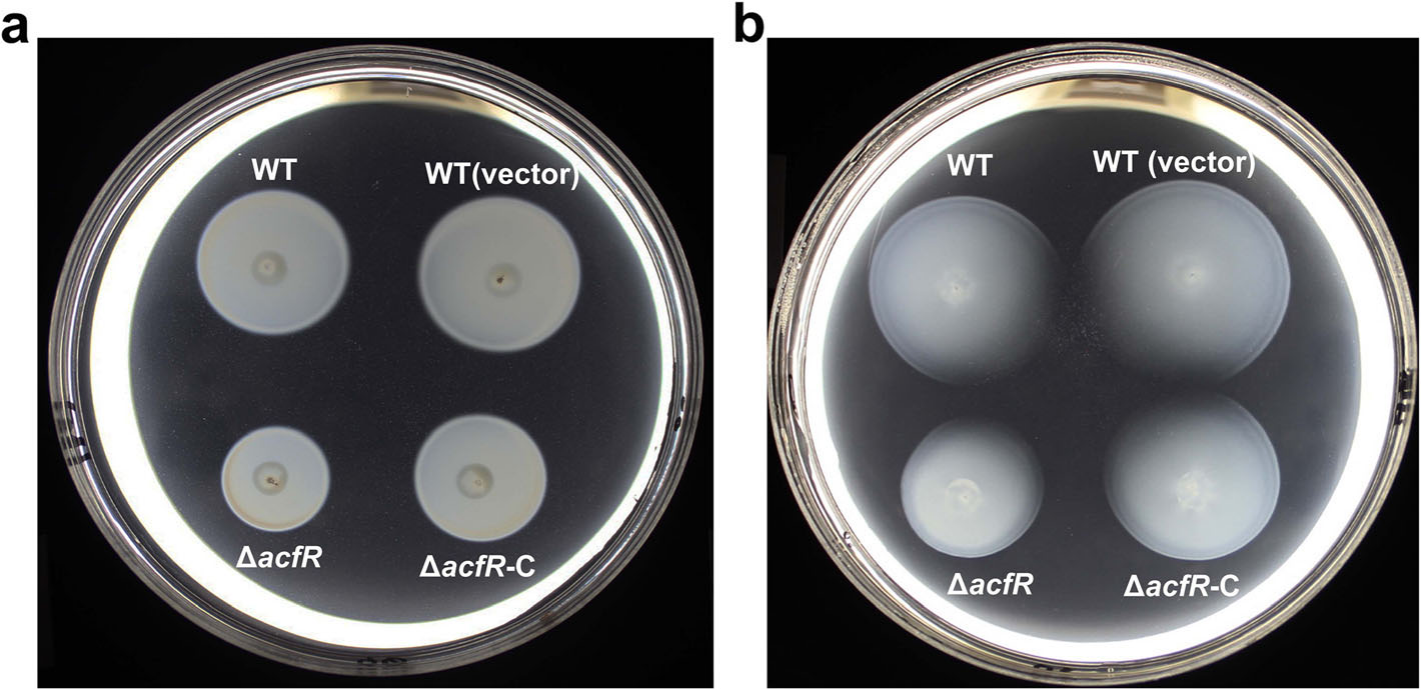
Mutation of *acfR* affects swimming motility ability. Swimming motility behaviour of the *A. caulinodans* (WT), WT containing pBBR1MCS-2 plasmid (WT vector), mutant (Δ*acfR*), and complemented strains (Δ*acfR*-C) were tested on 0.3% soft agar plates. Representative L3 plates with 10 mM sodium lactate (**a**) or glycine (**b**) as the sole carbon source are shown

To further investigate the functions of AcfR in bacterial motility behaviour, the swimming activity of the WT, mutant (Δ*acfR*), and complemented (Δ*acfR-C*) strains was tested on 0.3% soft agar plates. Bacterial cultures of these strains were inoculated in the middle of soft agar plates. The plates were incubated for 2–3 days at 37 °C. As shown in Fig. 4, the mutant strain exhibited decreased swimming motility ability compared to that of the WT on L3 plates, with sodium lactate (Fig. 4a) or glycine (Fig. 4b) serving as the sole carbon sources. The motility-deficient phenotype in the mutant was able to be rescued by the complemented strain Δ*acfR-C.* These results indicated that AcfR positively modulates the cells swimming motility behaviour of *A. caulinodans* ORS571.

**Fig. 5:**
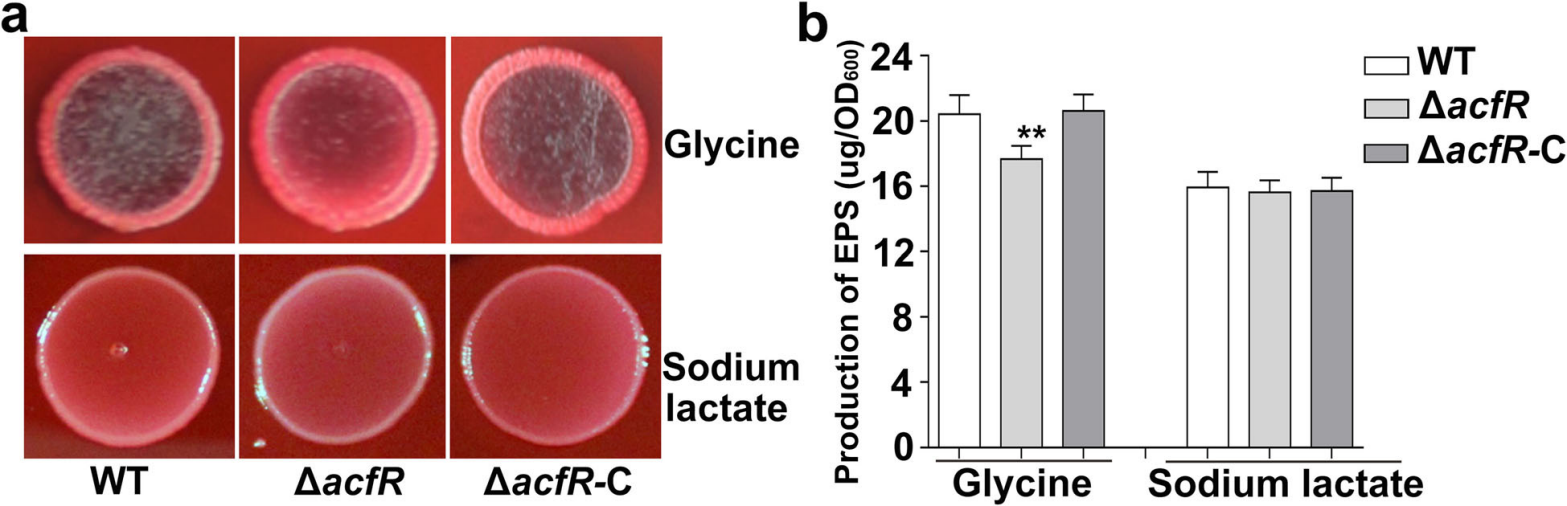
Morphotypes observation and quantitative analysis of EPS production. **a** The morphology of colonies (WT, Δ*acfR*, and Δ*acfR*-C) grown on L3 Congo red plates at 37 °C for 2–3 days. L3 plates contain 10 mM glycine or sodium lactate as the sole carbon source. **b** Quantitative analysis of the total EPS produced by strains of WT, Δ*acfR*, and Δ*acfR*-C. The error bars indicate standard deviations which were calculated by three independent experiments. Statistically significant differences between the WT and mutant strain was indicated by the asterisks (**P ≤ 0.01)

Congo red is often used to detect exopolysaccharide (EPS) production in bacteria [15]. Figure 5 shows that the EPS production levels of the WT, mutant, and complemented strains on Congo red plates. There were no significant differences in the colony morphology and total EPS production among the WT, Δ*acfR*, and Δ*acfR-*C strains when they were grown on Congo red plates with sodium lactate as the sole carbon source. However, the colony morphology and the total EPS production were significantly different (*P* ≤ 0.01) between the Δ*acfR* and WT cells. The Δ*acfR mutant* produced less “black pigmented” (Fig. 5a) and total EPS (Fig. 5b) than did the WT when grown on L3 medium containing glycine as the sole carbon source. These results indicated that AcfR was involved in the secretion process of EPS and that different carbon sources might influence the regulation of AcfR on the EPS phenotype.

### Δ*acfR* mutant strain is impaired in competitive nodulation of the host plants

**Fig. 6:**
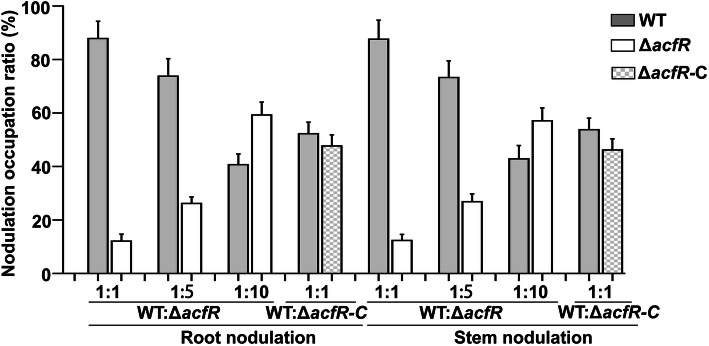
The Δ*acfR* mutant reduced nodulation competitiveness on roots and stems. The Δ*acfR* mutant was mixed at a 1:1, 1:5 or 1:10 with WT and inoculated onto roots and stems of *S. rostrata*. The complemented strain Δ*acfR*-C and WT was mixed at a 1:1 ratio. Data are shown the % of WT, mutant or complemented strain recovered from root nodules or stem nodules. At least 100 colonies that reisolated from randomly selected nodules from ten individual plants were identified and analyzed. The error bars represent the standard deviations of three independent experiments

To investigate whether the motility and EPS formation phenotypic defects of the Δ*acfR* mutant also affect symbiosis characteristics with the host plant, competitive nodulation assays were performed by analysing the levels of nodulation on the host plant. To test whether Δ*acfR* possessed a competitive disadvantage when competing with WT, cultures of WT and Δ*acfR* were mixed at 1:1, 1:5, and 1:10 ratios and subsequently incubated with *S. rostrata* roots and stems. The ratio of the number of wild-type cells to the number of mutant cells in the inoculum was determined by cell counts performed before mixing. At the end of each experiment (usually 35 days), the ratio of nodules induced by the wild-type or the mutant strain was determined by PCR to detect the colonies that grew from surface-sterilized crushed nodules on TY agar plates. The results shown in Fig. 6 demonstrate that the WT formed more nodules (by five- to sixfold) than the mutant strain when inoculated on roots and stems at a 1:1 ratio. With increases in the ratio of the inoculated mutant strain (WT:Δ*acfR* = 1:5 or 1:10), the nodule occupancy also increased. These results indicated that the competitive nodulation ability of the mutant strain was dramatically weakened compared with that of the WT. However, the number of nodules induced by Δ*acfR-*C was not significantly different compared with that of the WT when inoculated at a 1:1 ratio, indicating that the competitive nodulation ability was restored in the complemented strain. Therefore, we conclude that the AcfR regulator is essential for normal nodulation competitiveness during symbiosis on *S. rostrata*.

## Discussion

AcfR, a typical two-component response regulator, includes an N-terminal REC domain and a C-terminal HTH_LuxR DNA-binding domain. Previous studies have shown that among the 991 LuxR-associated protein sequences (containing at least one HTH_LuxR domain) of Actinobacteria, the REC domain appears to be associated with LuxR in 53% of cases [16]. These RECs present upstream LuxR domains and are primarily involved in the signal transduction pathway, suggesting that REC + HTH_LuxR proteins should be viewed not only as single transcription factors but also as response regulators of TCS. In some gram-positive bacteria of Actinobacteria, the REC + HTH_LuxR proteins are most commonly utilized as RRs of TCS. This specific association of REC + HTH_LuxR appears to be a widespread phenomenon in *B. subtilis* and *Lactobacillus plantarum* [17] that is involved in QS-related competence regulation.

In gram-negative bacteria, NarL with the REC + HTH_LuxR domain regulates the nitrate/nitrite signal transduction pathway in *E. coli* [18]. In *S. meliloti*, the FixJ and FixL are two-component regulators that are widely spread among prokaryotes. FixL, as a transmembrane protein, senses and transduces environmental signals to FixJ. Next, the transcriptional regulator FixJ activated the expression of *nif* and *fix* genes [19]. In the genome of the gram-negative bacterium *A. caulinodans* ORS571, seven REC + HTH_LuxR regulators were identified. Our previous results showed that the REC + HTH_LuxR regulator AclR1 (WP_012169103) is involved in the c-di-GMP signalling pathway and initiates a downstream related signal transcription pathway [20]. In this work, we have characterized the response regulator AcfR of *A. caulinodans*, which showed multiple affected phenotypes: (i) AcfR positively regulates the swimming motility behaviour of A. caulinodans under free-living conditions but does not affect the normal growth of cells; (ii) the *acfR* gene deletion mutant strain produced less “black pigmentation” and EPS than the WT when glycine was used as the sole carbon source; and (iii) AcfR positively regulated the competitive nodulation ability of *S. rostrata* roots and stems. These pleiotropic phenotypes were attributable to the deletion of the *acfR* gene, which encodes a REC + HTH_LuxR response regulator of the NarL/FixJ family. Rhizobial motility, chemotaxis and EPS are considered to play essential roles in the early stages of competitive colonization and nodulation [21, 22]. AcfR of *A. caulinodans* ORS571 regulates competitive nodulation ability and may directly affect the symbiotic pathway or indirectly regulate symbiosis by affecting bacterial motility behaviour and EPS production. Further research is warranted to investigate the signalling pathway of symbiosis between *A. caulinodans* and its host plant.

## Conclusions

Our results indicated that AcfR, as a FixJ family response regulator, is involved in the regulation of gram-negative bacterial motility, exopolysaccharide production and competitive symbiosis processes with the host. These findings indicate that AcfR is both a response regulator of the TCS and a transcription factor that plays an important role in regulating *Rhizobium*-legume host symbiosis.

## Methods

### Bacterial strains and growth conditions

*A. caulinodans* ORS571 was used throughout this study as the wild-type strain. All strains and plasmids used in this study are listed in Table 1. Bacteria (*E. coli*) were routinely grown overnight in Luria-Bertani (LB) broth or on LB agar at 37 °C for the amplification of recombinant clones and plasmid isolation. The *A. caulinodans* ORS571 WT strain and mutant Δ*acfR* were grown in L3 medium containing 100 μg/mL ampicillin and 25 μg/mL nalidixic acid antibiotics at 37 °C [22]. The complemented strain Δ*acfR-*C was grown in L3 medium containing 100 μg/mL ampicillin, 25 μg/mL nalidixic acid and 50 μg/mL kanamycin. The L3 medium was supplemented with 10 mM NH_4_Cl as nitrogen source and with 10 mM glycine or succinate as the sole carbon sources.

**Table 1:** Strains and plasmids used in the study

Strain or plasmid	Relevant properties	Source or reference
**Strain:**		
*A. caulinodans* ORS571	Wild-type strain, *Amp*^*r*^, *Nal*^*r*^	[1]
Δ*acfR*	ORS571 derivative, *acfR* deletion mutant, *Amp*^*r*^, *Nal*^*r*^	This study
Δ*acfR*-C	Δ*acfR* mutant harboring the pBBR-*acfR* plasmid, *Amp*^*r*^, *Nal*^*r*^, *Kan*^*r*^	This study
*E. coli* DH5a	*F*^*−*^S--upE44 Δ*lac*U169 (*φ80 lacZΔM15*) hsdR17 *rec*A1 *endA1 gyrA96 thi-1 relA1*	Transgen
**Plasmid:**		
pCM351	Mobilizable allelic exchange vector, *Amp*^*r*^, *Gen*^*r*^	[23]
pCM157	IncP plasmid that expresses Cre recombinase, *Tet*^*r*^	[23]
pRK2013	Helper plasmid, ColE1 replicon, Tra^+^, *Kan*^*r*^	[24]
pBBR1MCS-2	Broad-host-range cloning vector, *Kan*^*r*^	[25]
pBBR-*acfR*	pBBR1MCS-2 with *acfR* ORF and upstream promoter region, *Kan*^*r*^	This study

*Amp*^*r*^ ampicillin resistance, *Nal*^*r*^ Nalidixic acid, *Gen*^*r*^ gentamicin resistance, *Kan*^*r*^ kanamycin resistance, *Tet*^*r*^ tetracycline resistance

### Phylogenetic analysis

Amino acid sequences were aligned and analysed with the multiple sequence alignment program ClustalW of BioEdit software. Phylogenetic and molecular evolutionary analyses were conducted using MEGA-X version. A neighbour-joining phylogenetic tree was constructed by using amino acid sequences with 1000 bootstrap replications. These sequences include AcfR and NarL/FixJ family proteins (FixJ family response regulator of *Azorhizobium* sp. AG788, FixJ of *S. meliloti*, FixJ of *A. caulinodans*, NarL, CitB, RcsB, and UhpA). QS-LuxR of *Vibrio fischeri* as an outgroup was analysed to construct a phylogenetic tree.

### Construction of mutant, and complemented strains

To construct the *acfR* gene deletion mutant, a 710-bp fragment was amplified using the primers AcfRUF and AcfRUR (Table 2) and inserted into pCM351 [23] after restriction with KpnI and NdeI. The positive recombinant plasmid was designated pCM351::UF. A 650-bp fragment was amplified with primers AcfRDF and AcfRDR (Table 2) and cloned into the pCM351::UF after restriction with ApaI and Age1. The positive plasmid (pCM351::UF::DF) was transformed into ORS571 by using pRK2013 as the helper plasmid [24], and the *acfR* gene deletion mutant was subsequently screened based on homologous double exchange as previously described [26]. The correct integration of the mutants was confirmed by PCR and named Δ*acfR.*

**Table 2:** Oligonucleotides used in the study

Primer name	Sequence (5′-3′)^**a**^	Purpose
AcfR-UF-KpnI	GGGGTACCCTGATGACCCTCAGCCAGTTG	Δ*acfR* mutant construction
AcfR-UR-NdeI	TCCATATGTGGATCGTTTGACGAGGTCAC	Δ*acfR* mutant construction
AcfR-DF-ApaI	TCGGGCCCGAGACGCGTCGGAATTCCTGA	Δ*acfR* mutant construction
AcfR-DR-AgeI	CGACGCGTAATGGCGATGCCAACTACCGA	Δ*acfR* mutant construction
AcfR-CF-KpnI	GGGTACCTGGGTGCGACGCTA TCACGGA	Δ*acfR-*C construction
AcfR-CR-BamHI	CGGGATCCCTGACGGCAGACATCGAAACG	Δ*acfR-*C construction
AcfR-F	GTGACCTCGTCAAACGATCCA	Validation of *acfR*
AcfR-R	TCAGGAATTCCGACGCGTCTC	Validation of *acfR*

^a^Engineered restriction sites are underlined

To construct the complemented strain of Δ*acfR*, a fragment containing the *acfR* gene and the predicted promoter sequence was obtained by PCR with the primer combination AcfR-CF/AcfR-CR and subsequently was digested with KpnI and BamHI. This fragment was ligated into the vector pBBR-MCS2 [25], which had been cut with KpnI and BamHI. The correct sequence was verified by DNA sequencing. Next, the recombinant vector was transformed into the Δ*acfR* mutant to screen the complemented strain (Δ*acfR*-C) based on kanamycin resistance.

### Growth kinetics experiments

The growth kinetics of WT and mutant strains were grown in TY medium with 25 μg/ml nalidixic acid and 100 μg/ml ampicillin antibiotics. The overnight cultures were diluted into fresh L3 medium to an initial OD_600_ of 0.02 and grown at 37 °C with rotary shaking at 180 rpm. Growth kinetics were determined by monitoring turbidity at 600 nm. The experiments were performed in triplicate, and the data shown are the means and standard deviation.

### Motility behaviour assays

Motility assays were performed on 0.3% soft agar L3 plates according to previous publications [26]. The L3 plates contained a 10 mM carbon source (sodium lactate or glycine) and 10 mM NH_4_Cl. Briefly, 5 μl of overnight grown cultures were added to plates and incubated at 37 °C for 2–3 days. The swimming diameter at the agar surface was utilized to assess the swimming motility ability.

### Exopolysaccharide production assays

L3 plates supplemented with 40 μg/ml Congo red, 10 mM NH_4_Cl and 10 mM carbon sources (sodium lactate and glycine) were used to determine the Congo red-binding properties of colonies. To examine EPS production, mid-logarithmic phase cultures were adjusted at an OD_600_ of 1.0, and 15 μl bacterial cultures were spotted onto the L3 plates (0.8% agar). Morphological observations were made after an incubation period of 2–3 days at 37 °C. The total EPS was quantified by referring to the method described by Liu et al. [20].

### Competitive nodulation assays on plant roots

Plant cultivation and nodulation tests were performed as described by previously [27, 28] with minor modifications. Briefly, two types of treatment were tested: WT:Δ*acfR* at approximately 1:1, 1:5 and 1:10 and WT:Δ*acfR*-C in approximately 1:1. For competitive nodulation on roots, surface-sterilized seedlings of *S. rostrata* were inoculated with bacterial cultures corresponding to each treatment at an OD_600_ of 0.5 for 30 min. For competitive nodulation on stems, cell suspensions corresponding to each treatment were painted onto *S. rostrata* stems after 35 days of plant growth. Ten plants were used for each of the three treatments. Bacterial endophytes were then isolated from surface-sterilized root or stem nodules at 35 days postinoculation and analysed. The WT and Δ*acfR* reisolated from nodules were determined by PCR using the primer pair AcfR-F and AcfR-R. The WT and Δ*acfR*-C reisolated from nodules were distinguished based on kanamycin resistance markers.

### Statistical analysis

All statistical analyses were performed using the SPSS 17.0 software package. We determined average values from at least three independent experiments and performed one-way analysis of variance followed by pairwise two-sample t-test assuming equal variances. Student’s t-test assuming equal variances was used to calculate the *p*-values. *P*-values < 0.05 and < 0.01 were tested. Each experiment was repeated in at least three independent experiments.

## Supplementary Information

**Additional file 1: Figure S1.** Domain architecture and interaction network of AcfR. (a) Domain architecture of AcfR predicted by SMART. AcfR was identified encoding a protein of 214 amino acids that containing a REC domain and a HTH_LuxR domain. (b) The interactive protein network of the AcfR predicted by STRING. Eight proteins (containing two AAA family ATPase, two PAS domain S-box, FixL, etc.) predicted to interact with *A. caulinodans* AcfR. (c) The predicted functional partners based on the neighborhood evidence, cooccurrence evidence, and text-mining evidence. **Figure S2.** Domain structures of proteins that predicted in Fig. S1. The protein domains were predicted by using the SMART program. There are eight predicted interaction proteins (AZC_0278, 2411, 2412, 3126, 3970, 3971, 3914, and 0489). Six of eight proteins with HATPase_c and HisKA domains. Abbreviations: PAS, Per-Arnt-Sim domain; PAC, Motif C-terminal to PAS motifs; GAF, Domain present in phytochromes and cGMP-specific phosphodiesterases; HATPase_c, Histidine kinase-like ATPases; HisKA, His Kinase A (phospho acceptor) domain. **Figure S3.** Growth rates of the wild-type, mutant, and complemented strain are similar. Growth curves of the WT, Δ*acfR*, and Δ*acfR*-C in L3 liquid medium with 10 mM sodium lactate as sole carbon source and 10 mM NH4Cl as nitrogen source.

## Data Availability

The datasets used and/or analyzed during the current study available from the corresponding author on reasonable request.

## Abbreviations

TCS: Two-component regulatory system
HK: Histine kinase
RR: Response regulator
REC: Receiver domain
HTH: Helix-turn-helix
QS: Quorum sensing
HATPase_c: Histidine kinase-like ATPases
HisKA: His Kinase A domain
ORF: Open reading frame
EPS: Extracellular polysaccharide
