# Supplementary material for: FixJ family regulator AcfR of Azorhizobium caulinodans is involved in symbiosis with the host plant
Source: BMC Microbiol. 2021 Mar 9;21:80. doi: 10.1186/s12866-021-02138-w (PMC7945327; doi:10.1186/s12866-021-02138-w)
Supplement: Supplementary file 1 — Additional file 1: Figure S1. Domain architecture and interaction network of AcfR. (a) Domain architecture of AcfR predicted by SMART. AcfR was identified encoding a protein of 214 amino acids that containing a REC domain and a HTH_LuxR domain. (b) The interactive protein network of the AcfR predicted by STRING. Eight proteins (containing two AAA family ATPase, two PAS domain S-box, FixL, etc.) predicted to interact with A. caulinodans AcfR. (c) The predicted functional partners based on the neighborhood evidence, cooccurrence evidence, and text-mining evidence. Figure S2. Domain structures of proteins that predicted in Fig. S1. The protein domains were predicted by using the SMART program. There are eight predicted interaction proteins (AZC_0278, 2411, 2412, 3126, 3970, 3971, 3914, and 0489). Six of eight proteins with HATPase_c and HisKA domains. Abbreviations: PAS, Per-Arnt-Sim domain; PAC, Motif C-terminal to PAS motifs; GAF, Domain present in phytochromes and cGMP-specific phosphodiesterases; HATPase_c, Histidine kinase-like ATPases; HisKA, His Kinase A (phospho acceptor) domain. Figure S3. Growth rates of the wild-type, mutant, and complemented strain are similar. Growth curves of the WT, ΔacfR, and ΔacfR-C in L3 liquid medium with 10 mM sodium lactate as sole carbon source and 10 mM NH4Cl as nitrogen source. [file 12866_2021_2138_MOESM1_ESM.pdf]

FixJ family regulator AcfR of *Azorhizobium caulinodans* is involved in symbiosis with the host plant

Wei Liu[a, b, c], Xue Bai[a], Yan Li[a, b, c], Haikun Zhang[a, b, c], Xiaoke Hu[a, b, c*]

[a]Key Laboratory of Coastal Biology and Bioresource Utilization, Yantai Institute of Coastal Zone Research, Chinese Academy of Sciences, Yantai, 264003, China

[b]Laboratory for Marine Biology and Biotechnology, Qingdao National Laborator y for Marine Science and Technology, Qingdao, 266237, China

[c]Center for Ocean Mega-Science, Chinese Academy of Sciences, Qingdao, China

*Correspondence: xkhu@yic.ac.cn

E-mail addresses: wliu@yic.ac.cn; 838208457@qq.com; liyan@yic.ac.cn; hkzhang@yic.ac.cn

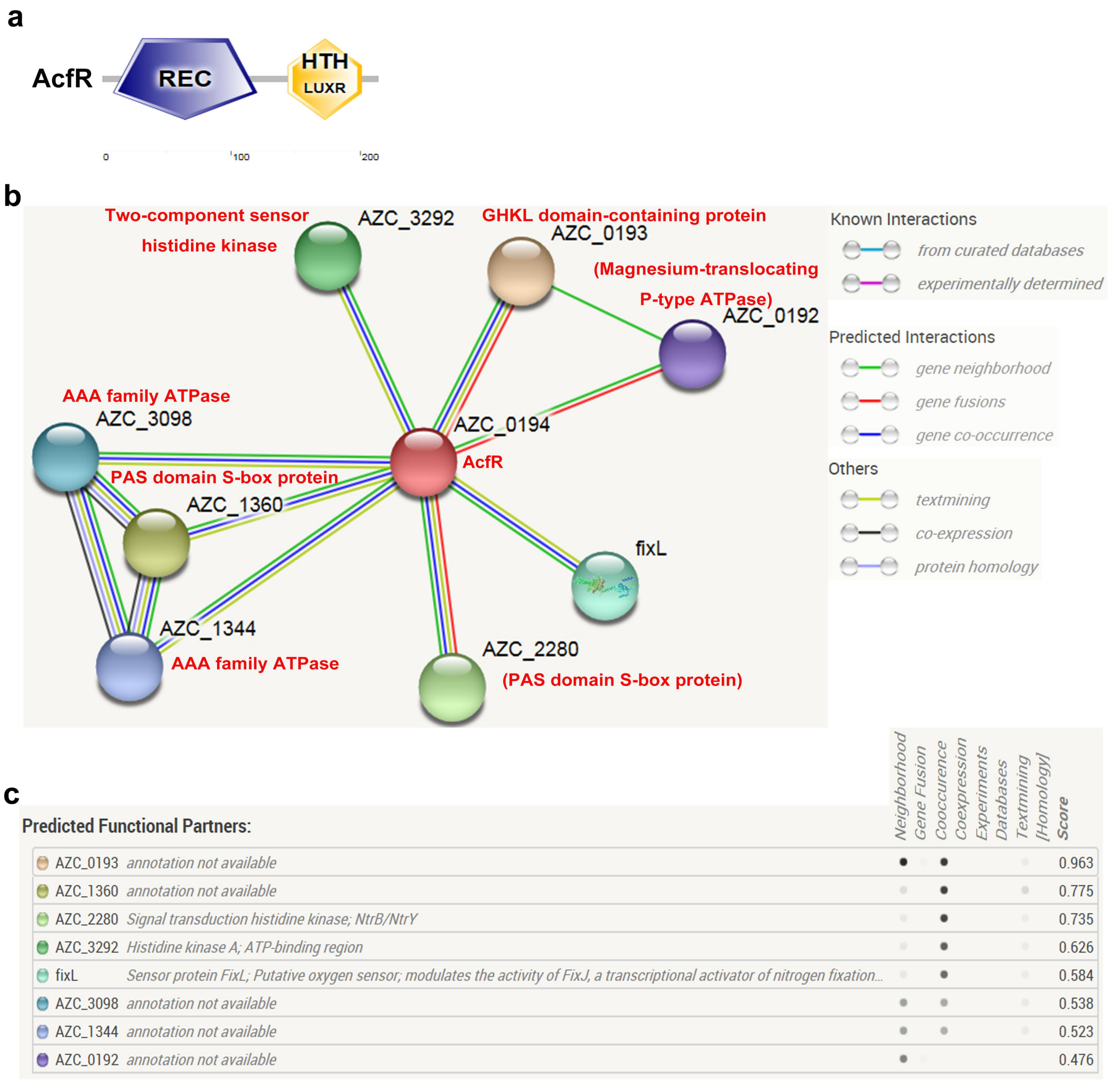

Fig. S1 Domain architecture and interaction network of AcfR. (a) Domain architecture of AcfR predicted by SMART. AcfR was identified encoding a protein of 214 amino acids that containing a REC domain and a HTH_LuxR domain. (b) The interactive protein network of the AcfR predicted by STRING. Eight proteins (containing two AAA family ATPase, two PAS domain S-box, FixL, etc.) predicted to interact with *A. caulinodans* AcfR. (c) The predicted functional partners based on the neighborhood evidence, cooccurrence evidence, and text-mining evidence.

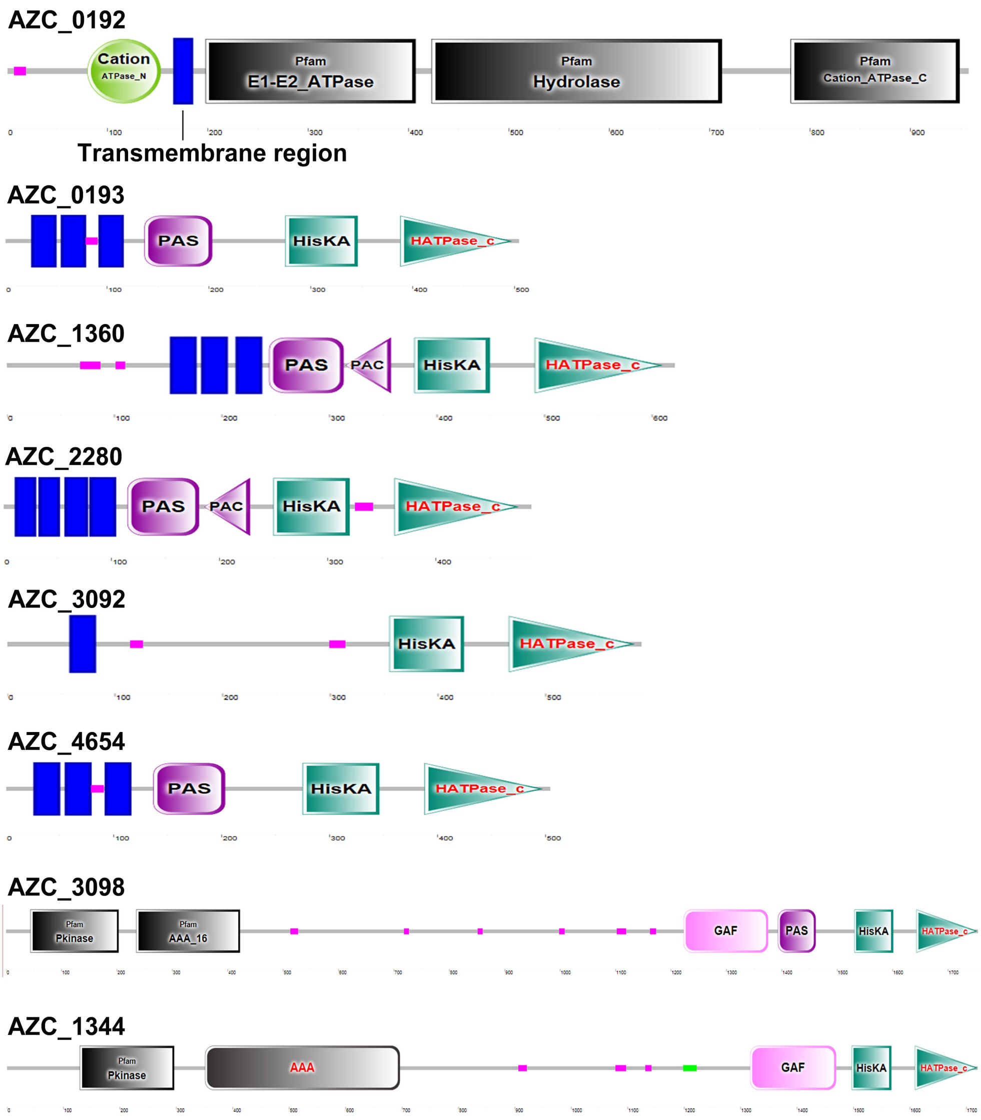

Fig. S2 Domain structures of proteins that predicted in Fig. S1. The protein domains were predicted by using the SMART program. There are eight predicted interaction proteins (AZC_0278, 2411, 2412, 3126, 3970, 3971, 3914, and 0489). Six of eight proteins with HATPase_c and HisKA domains. Abbreviations: PAS, Per-Arnt-Sim domain; PAC, Motif C-terminal to PAS motifs; GAF, Domain present in phytochromes and cGMP-specific phosphodiesterases; HATPase_c, Histidine kinase-like ATPases; HisKA, His Kinase A (phospho acceptor) domain.

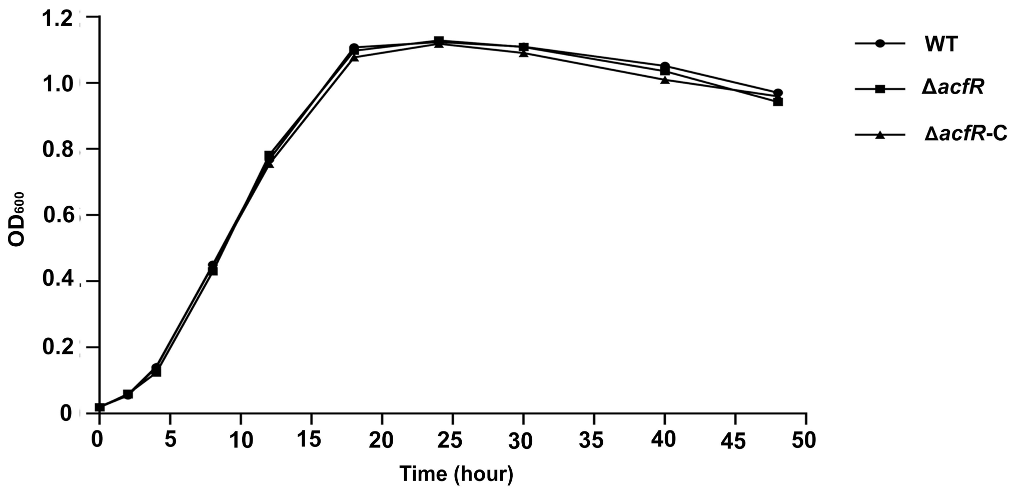

Fig. S3 Growth rates of the wild-type, mutant, and complemented strain are similar. Growth curves of the WT, Δ*acfR*, and Δ*acfR*-C in L3 liquid medium with 10 mM sodium lactate as sole carbon source and 10 mM NH_4Cl as nitrogen source.
